# Build me up to break me down: mapping grain development and germination in barley over time and space

**DOI:** 10.1093/plcell/koag130

**Published:** 2026-05-04

**Authors:** Pablo González-Suárez, Alexa-Maria Wangler

**Affiliations:** Assistant Features Editor, The Plant Cell, American Society of Plant Biologists; Department of Developmental Genetics, Centre for Plant Molecular Biology (ZMBP), Eberhard Karls University, Tuebingen D-72076, Germany; Department of Developmental Genetics, Centre for Plant Molecular Biology (ZMBP), Eberhard Karls University, Tuebingen D-72076, Germany

Many flowering plants rely on seeds for reproduction and dispersal. To maximize the next generation's chances of survival, seed set and germination must be precisely timed, particularly in annual plants that only reproduce once. Given the sheer importance of seeds for food security, it is no surprise that their development and germination are among the most studied processes in plant biology. However, seeds contain tissue layers of distinct genome compositions (Fig. 1a), which complicates developmental studies at the cell-specific level. Recently, spatial transcriptomics has emerged as a valuable tool to overcome this, allowing researchers to profile gene expression without losing native spatial information. Still, studies that profile spatiotemporal gene expression during seed development remain limited, particularly in crops (Fu et al. 2023; Peirats-Llobet et al. 2023; Yao et al. 2024).

**Figure 1:**
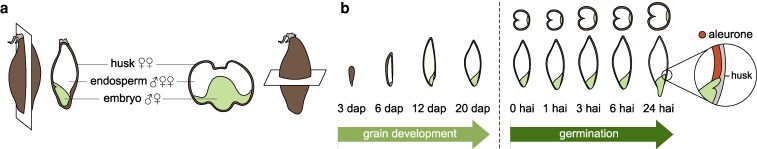
Building a spatiotemporal transcriptional atlas of barley grain development and germination. a) The barley grain is a genetically complex structure that includes the diploid maternal tissue, a triploid endosperm, and the diploid embryo. b) Using longitudinal and transverse sections of barley grains at different timepoints, the authors generated a spatial transcriptomic atlas of grain development and germination. The atlas allows to interrogate specification and development of distinct tissues such as the aleurone layer. Figure credit: P. González-Suárez.

In their recent work, **Marta Peirats-Llobet and colleagues** (Peirats-Llobet et al. 2026) set out to characterize transcriptomic changes during grain development and germination in barley (*Hordeum vulgare*). Leveraging spatial transcriptomics, they constructed a rich transcriptomic atlas of barley grains. By performing multiple parallel sections of seeds in both longitudinal and transversal orientations, they obtained 3-dimensional information of gene expression across different tissues. Additionally, they sampled a total of 9 timepoints spanning both seed set and germination, resulting in a final dataset that captured multiple key developmental stages (ie syncytial, cellularization, early and late storage, imbibition, and stationary) (Fig. 1b).

Further integration of the 3-dimensional data from different timepoints enabled the creation of a 4-dimensional atlas that renders transcript kinetics for different seed tissues. Using known markers, the authors were able to annotate spatial clusters and assign them to distinct cell types present during seed development and germination. Interestingly, many genes exhibited mottled expression within and between clusters, while housekeeping genes such as *Actin* and *Tubulin* remained homogenous, hinting at heterogeneous gene expression patterns across tissues during seed development.

The new dataset allows researchers to examine the transcriptional dynamics of a plethora of genes involved in seed development and germination. Among others, the authors highlight spatiotemporal changes in the expression of genes involved in energy biology, including starch metabolism and sugar transport. Starch is a major component of cereal grains and has a prominent nutritional value, making the investigation of its metabolism an agricultural priority. The atlas can be used not only to advance our genetic understanding of agronomically relevant traits but also to explore developmental mechanisms. Given the central role of plant hormones in grain development and germination, the authors examined changes in transcripts related to metabolism and signaling of ABA, gibberellic acid, brassinosteroids, and auxin. In particular, they found heterogeneous expression patterns of auxin-related genes and auxin transporters during seed set and germination, which may inform future research investigating how hormonal gradients shape grain development.

From a cell biology perspective, the transcriptomic atlas provides a valuable tool to inspect changes in cell identity during grain and early seedling development. Specifically, the authors focus on the aleurone, a specialized tissue derived from the outermost layer of the endosperm and involved in the breakdown of starch reserves to nurture the growing seedling. Pseudotime and coexpression analyses of aleurone lineage cells recapitulate the observed changes in gene expression with high accuracy and pinpoint key processes involved in aleurone cell development. The inferred trajectory of aleurone lineage cells reveals spatiotemporal dynamics of regulators previously linked to aleurone development. Beyond the aleurone, the atlas can also help identify novel genes involved in precise aspects of cereal grain development and germination.

The 4-dimensional atlas curated by Peirats-Llobet et al. (2026) presents an invaluable resource to investigate grain development in barley. Through the web interface (https://barley-4d.latrobe.edu.au/or https://barley-4d-gene-atlas.hutton.ac.uk/), genes of interest can be queried to identify transcript dynamics over time and space. Furthermore, in contrast to previous research in cereals (Fu et al. 2023; Yao et al. 2024), the study spans both seed development and germination, allowing cell lineages to be followed from specification through to differentiation. Taken together, these resources provide a unique tool for studying the developmental control of seed biology in barley and related cereals.

## Recent related articles in *The Plant Cell*:

- Long et al. (2025) used spatial transcriptomics to profile gene expression along the apical-basal axis during wheat spikelet development.
- Sami et al. (2025) utilized publicly available RNA-seq data to examine the phylotranscriptome during Arabidopsis seed development, uncovering a reverse hourglass pattern during seed maturation.
- Kovacik et al. (2024) performed bulk RNA-seq on dissected barley grain compartments, including embryo, endosperm, and maternal tissues across different developmental stages.

## Data Availability

No new data were generated or analysed in support of this research.
